# Microencapsulation of sea buckthorn (*Hippophae rhamnoides* L.) pulp oil by spray drying

**DOI:** 10.1002/fsn3.1828

**Published:** 2020-10-22

**Authors:** Sining Xu, Zhishu Tang, Hongbo Liu, Mei Wang, Jing Sun, Zhongxing Song, Chunli Cui, Chen Sun, Shijun Liu, Zheng Wang, Jingao Yu

**Affiliations:** ^1^ Shaanxi Collaborative Innovation Center of Chinese Medicinal Resources Industrialization Shaanxi University of Chinese Medicine Xianyang China; ^2^ Shaanxi Province Key Laboratory of New Drugs and Chinese Medicine Foundation Research Xianyang China; ^3^ Affiliated Hospital, Shaanxi University of Chinese Medicine Xianyang China; ^4^ Shaanxi Xingshengde Pharmaceutical Limited Liability Company Tongchuan China; ^5^ College of Pharmacy Shaanxi University of Chinese Medicine Xianyang China

**Keywords:** gum arabic, maltodextrins, microencapsulation, sea buckthorn pulp oil, spray drying

## Abstract

The aim of this work was to encapsulate sea buckthorn (*Hippophae rhamnoides* L.) pulp oil (SBPO) by spray drying. Gum Arabic (GA) and maltodextrins (MD) were used as wall materials. The effects of several factors, including GA to MD ratio, total solids content of emulsion, wall to core ratio, and inlet air temperature, on the microencapsulation efficiency (ME) were investigated. The optimization of operation conditions was realized by response surface methodology (RSM). The optimal conditions were as follows: GA to MD ratio 2.38, total solids content 39%, wall to core ratio 5.33, and inlet air temperature 154°C. Under the optimal conditions, the ME of SBPO microcapsules was 94.96 ± 1.42%. The physicochemical properties of microcapsules were also invested. SBPO microcapsules obtained had low water activity, low moisture content, high water solubility, and high bulk density. For the morphological characteristics, cracks and pores were not observed in most microcapsules, which was beneficial for the protection of ingredients in microcapsules. The particle size of SBPO microcapsules ranged from 0.01 to 5 μm, and the mean diameter *d*_4,3_ was 2.55 μm. The analysis results of fourier transform infrared spectroscopy (FTIR) informed the presence of SBPO in microcapsules. There were no significant differences in the content of the main fatty acids in SBPO before and after spray drying. The results of oxidative stability showed that the microencapsulation by spray drying could effectively protect SBPO from oxidation and extend the shelf life of SBPO.

## Graphical abstract

Spray‐drying technology was used to encapsulate sea buckthorn (*Hippophae rhamnoides* L.) pulp oil, and the effects of main factors were investigated. As well as, the physical properties of microcapsules including water activity, moisture content, solubility, hygroscopicity, morphology, and particle size were also studied. Under the optimal conditions, the microencapsulation efficiency and qualities of microcapsules were satisfactory.

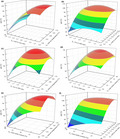

## 1 INTRODUCTION

Sea buckthorn (*Hippophae rhamnoides* L.) is a valuable plant resource for both medicine and food, which is widely distributed in the temperate zone of the Eurasian continent, such as Germany, France, India, China, and Russia. The health benefits of sea buckthorn had been recorded by classic medical works including the Sibu Yidian (Tang Dynasty), the Ben Cao Gang Mu (Ming Dynasty), and the Jing Zhu Ben Cao (Qing Dynasty). It is a great valuable plant for its nutritional and medicinal potential (Bal, Meda, Naik, & Satya, 2011; Suryakumar & Gupta, 2011; Zeb, 2004a). Almost all parts of sea buckthorn, including pulps, seeds, leaves, and bark, are an abundant source of lots of bioactive substances such as vitamins, lipids, essential amino acids, carotenoids, organic acids, unsaturated fatty acids, tannins, phytosterols, and flavonoids (Li & Schroeder, 1996; Tiitinen, Hakala, & Kallio, 2005).

Sea buckthorn pulp oil (SBPO), obtained from the pulp of sea buckthorn berries, also is a kind of very valuable edible and medical oil. It has rich nutritional and medicinal components (Dulf, 2012; Fatima et al., 2012; Olas, 2016, 2018; Teleszko, Wojdylo, Rudzińska, Oszmiański, & Golis, 2015; Yang, Karlsson, Oksman, & Kallio, 2001; Zielińska & Nowak, 2017), including fatty acids (palmitoleic acid, oleic acids, linoleic acid, palmitic acid and so on), lipids (phospholipids, glycolipids and etc.), vitamins (vitamin A, B, C, E, F, and P), phytosterols (β‐sitosterol, Δ^5^‐avensterol, Δ^7^‐avenastenol, stigmasterol, campesterol, citrostadienol, and etc.), flavonoids (kaempferol, quercetin, isorhamnetin, and etc.), carotenoid, fruit acids, and phenolic compounds..

Nowadays, SBPO has been used in food industry (Bal et al., 2011; Yang & Kallio, 2002; Zeb, 2004b), used in cosmetic industry as a component of skin care product, cream, and shampoo (Bal et al., 2011; Manea, Ungureanu, & Meghea, 2014; Staňková, Kremmyda, Tvrzická, & Žák, 2013; Zielińska & Nowak, 2017), and used in medicine industry as drug having significant therapeutic effect on antioxidant (Zielińska & Nowak, 2017), immunomodulatory (Yang et al., 1999), hepatoprotective activity (Czaplicki, Ogrodowska, Zadernowski, & Konopka, 2017), and cardioprotective (Eccleston et al., 2002; Johansson, Korte, Yang, Stanley, & Kallio, 2000; Suchal et al., 2016). However, SBPO is easily degraded when exposed to oxygen, light, water, and heat. Finding technologies to overcome these problems is desired. Microencapsulation is the process in which the substances that need to be protected often called core substances are wrapped by other substances often called wall materials to making the protected substances isolating from the surrounding environment (Kandansamy & Somasundaram, 2012). So microencapsulation is a good strategy to protect unstable substances from environmental factors. After microencapsulation, the oil is not only protected, but also its states changed from liquid to solid, which is useful of product processing.

Spray drying is a very popular microencapsulation technology employed in practical applications due to its low cost, agility, simple equipment and products with high quality (Gharsallaoui, Roudaut, Chambin, Voilley, & Saurel, 2007; Jafari, Assadpoor, He, & Bhandari, 2008; Pitalua, Jimenez, Vernon‐Carter, & Beristain, 2010). In order to obtain fine microcapsules, the main factors to be considered included the nature of wall materials, specifications of feed, and conditions of the spray drying.

It is because of the excellent film‐forming and emulsion properties of Gum Arabic (GA), making GA as a wall material widely used in the oil microencapsulation process (Bertolini, Siani, & Grosso, 2001; Fang, Shima, & Adachi, 2005; Jafari et al., 2008; Rosenberg, Kopelman, & Talmon, 1990). However, its high cost, limited supply, has restricted the use of GA. Maltodextrin (MD) can ameliorate the oxidation resistance of encapsulated oil but have the disadvantage of poor emulsion stability and low microencapsulation efficiency (ME) (Gharsallaoui, Saurel, & Chambin, 2012). The combination of GA and MD had been applied to the encapsulation of bitter melon extract (Tan, Kha, Parks, Stathopoulos, & Roach, 2015), soy oil (McNamee, O’Riordan, & O’Sullivan, 2001), fatty acids (Minemoto, Hakamata, Adachi, & Matsuno, 2002), and flavor (Apintanapong & Noomhorm, 2003). These results had shown that the combination of GA and MD could give play to their respective characteristics and make up each other's shortcomings, which reduced costs and improved product quality.

In this work, spray‐drying technology was used to encapsulate SBPO and made the GA and MD as the wall materials. The effects of main factors such as GA to MD ratio, a total solids content of the feed, wall to core ratio, and inlet air temperature on the ME were investigated. As well as, the physicochemical properties of microcapsules including water activity, moisture content, solubility, hygroscopicity, bulk density, morphology, particle size, fatty acids, and peroxide stability were also studied.

## 2 MATERIALS AND METHODS

### 2.1 Materials

SBPO was obtained from Shaanxi Guanchen Biological Technology Co., Ltd. GA was obtained from Tianjin Tianda chemical reagent Co., Ltd. MD was obtained from Shanghai Yuanye Biological Technology Co., Ltd. All other reagents were analytical purity.

### 2.2 Microencapsulation process

The microencapsulation process involved three basic steps as follows:

Preparation of emulsion. The mixture solution of GA and MD was prepared by dissolving them in double‐distilled water with constantly agitating (600 rpm) at a constant temperature 50 ± 1°C overnight. Then, 30 g of SBPO and 0.60 g of tween 20 surfactant were mixed gradually.

Homogenization of emulsion. Then the mixture was homogenized by using a dispersing machine (FJ‐200, Shanghai Specimen Company) at 5,590 *g* for 10 min to form a fine and stable O/W emulsion. The stability of emulsion was determined according to the method described by Tonon, Pedro, Grosso, and Hubinger (2012). The percentage of separation of emulsion under different experimental conditions all less than 5% indicated that the emulsion had good stability.

Spray‐drying emulsion. The O/W emulsion was sprayed by a YC‐1500 laboratory spray dryer with a 0.70 mm diameter rotating atomizer (Yacheng experimental Co. Ltd.). In order to keep homogeneous and stability, the emulsion was stirred constantly by a magnetic stirrer (SHJ‐3, Shuangjie Experimental Instrument Factory) at a constant temperature 50 ± 1°C during the entire process. The inlet temperature was set to test temperature. The pressure of compressed air was set to 0.2 MPa. The microcapsules were harvested and stored in 4°C fridge.

### 2.3 Single‐factor experiments

The effects of GA to MD ratio, total solids content of emulsion, wall to core ratio, and inlet air temperature were studied by a single‐factor design, and the ME was taken as evaluation index.

### 2.4 Experimental design of response surface methodology (RSM)

According to the single‐factor experimental results, a Box–Behnken design (BBD) with four independent variables was applied to optimize the process conditions. Each independent variable was coded: −1, 0, and +1. Four parameters, including GA to MD ratio, total solids content of emulsion, wall to core ratio, and inlet air temperature, were designated as *X*_1_, *X*_2_, *X*_3_, and *X*_4_, respectively. ME (*Y*) was taken as the corresponding value. Table 1 summarized the scope and level of the independent variables. A quadratic polynomial was fitted by regression analysis:

(1) $$Y=\beta_{0}+\sum_{j=1}^{\kappa }\beta_{j}X_{j}+\sum_{j=1}^{k}\beta_{\mathrm{jj}}X_{j}^{2}+\sum \sum_{i<j}\beta_{\mathrm{ij}}X_{i}X_{j}\;\left(k=4\right).$$

**TABLE 1:** BBD and response values for ME

Runs	*X*_1_	*X*_2_ (%)	*X*_3_	*X*_4_ (°C)	ME (%)	Predicted value
1	0 (2.3)	0 (30)	0 (4)	0 (150)	94.45 ± 1.11	93.01
2	0 (2.3)	0 (30)	1 (6)	1 (160)	94.32 ± 0.53	94.52
3	1 (4)	−1 (20)	0 (4)	0 (150)	82.23 ± 1.87	81.43
4	0 (2.3)	1 (40)	−1 (2)	0 (150)	64.00 ± 1.26	63.27
5	1 (4)	0 (30)	0 (4)	1 (160)	82.10 ± 1.89	81.49
6	0 (2.3)	0 (30)	0 (4)	0 (150)	92.66 ± 0.75	93.01
7	1 (4)	0 (30)	1 (6)	0 (150)	87.06 ± 1.58	86.35
8	0 (2.3)	−1 (20)	−1 (2)	0 (150)	37.72 ± 1.13	40.34
9	0 (2.3)	1 (40)	0 (4)	1 (160)	91.85 ± 1.53	90.76
10	−1 (0.6)	0 (30)	1 (6)	0 (150)	90.12 ± 0.74	90.17
11	0 (2.3)	1 (40)	1 (6)	0 (150)	95.34 ± 1.77	95.12
12	0 (2.3)	0 (30)	−1 (2)	1 (160)	52.40 ± 0.98	55.41
13	0 (2.3)	−1 (20)	0 (4)	−1 (140)	71.95 ± 1.36	70.08
14	0 (2.3)	1 (40)	0 (4)	−1 (140)	88.70 ± 1.81	86.96
15	0 (2.3)	0 (30)	−1 (2)	−1 (140)	42.53 ± 1.06	42.89
16	0 (2.3)	−1 (20)	0 (4)	1 (160)	85.02 ± 1.61	83.80
17	−1 (0.6)	0 (30)	−1 (2)	0 (150)	40.05 ± 0.25	37.80
18	0 (2.3)	0 (30)	0 (4)	0 (150)	93.15 ± 1.50	93.01
19	0 (2.3)	−1 (20)	1 (6)	0 (150)	91.08 ± 1.54	94.21
20	0 (2.3)	0 (30)	0 (4)	0 (150)	91.03 ± 1.55	93.01
21	−1 (0.6)	−1 (20)	0 (4)	0 (150)	66.82 ± 1.24	64.95
22	1 (4)	0 (30)	0 (4)	−1 (140)	74.50 ± 0.65	77.20
23	−1 (0.6)	1 (40)	0 (4)	0 (150)	86.30 ± 1.81	87.65
24	−1 (0.6)	0 (30)	0 (4)	1 (160)	80.56 ± 0.57	80.27
25	1 (4)	1 (40)	0 (4)	0 (150)	80.15 ± 1.13	82.57
26	0 (2.3)	0 (30)	0 (4)	0 (150)	93.75 ± 1.33	93.01
27	1 (4)	0 (30)	−1 (2)	0 (150)	56.02 ± 1.41	53.01
28	−1 (0.6)	0 (30)	0 (4)	−1 (140)	64.01 ± 1.53	67.02
29	0 (2.3)	0 (30)	1 (6)	−1 (140)	91.95 ± 1.48	89.50

In this fitting equation, *Y* was the response value; *β*_0_ represented a constant; *β_j_*, *β_jj_*, and *β_ij_* were the linearity, square, and interaction, respectively. *X_j_* and *X_i_* were the variables. *κ* was the number of variables.

### 2.5 Determination of ME

#### 2.5.1 Content determination of surface oil

4 g of microcapsules were placed in a breaker and added 40 ml hexane. The sample was kept a vortex movement for 1 min using a vortex apparatus (SBS100‐2, Select BioProducts). The solvent mixture was filtered into an evaporating dish. Triplicate determinations were performed. All solvents were collected and evaporated. The surface oil content was weighted.

#### 2.5.2 Content determination of total oil

The method was referred to as Partanen's method (Partanen, Yoshii, Kallio, Yang, & Forssell, 2002). 4 g of microcapsules were placed in a beaker and added 20 ml of 60°C water. The sample was then mixed with 2 ml of ammonia (25%) and stirred for 30 min at 50°C. Then, the 10 ml ethanol and 25 ml diethyl ether were added in order and gently shaken for 5 min. Finally, 25 ml petroleum ether was added and gently shaken for 5 min. The sample was kept still in some minutes, and phase separate on was allowed to occur. The upper organic phase was collected, and the lower aqueous phase was re‐extracted using ethanol, diethyl ether, and petroleum ether as the above method. Triplicate determinations were performed. All upper phase was collected in the evaporating dish, and the solvent was evaporated. The total oil content was weighted.

ME was calculated using the following formulas:

(2) $$\text{ME}\left(\%\right)=\frac{\text{Total}\;\text{oil}‐\text{Surface}\;\text{oil}}{\text{Total}\;\text{oil}}\times 100.$$

### 2.6 Properties of microcapsules

#### 2.6.1 Water activity and Moisture content

A water activity meter (LabMaster‐aw) was used to measure water activity with controlled temperature of 25 ± 0.5°C. The gravimetric method (AOAC, 1996) was applied for the determination of moisture content at 105°C.

#### 2.6.2 Water solubility index (WSI)

The measurement of WSI was referred to as Tan's method (Tan et al., 2015). The 50 ml of distilled water was mixed with 3 g of microcapsules and vigorously agitated for 30 min. Then, the supernatant obtained by the high‐speed centrifugation was dried at 105°C until the constant weight. WSI was calculated as followed:

(3) $$\text{WSI}\;\left(\%\right)=\frac{\text{Total}\;\text{solids}\;\text{in}\;\text{the}\;\text{supernatant}}{\text{The}\;\text{weight}\;\text{of}\;\text{microcapsules}}\times 100.$$

#### 2.6.3 Hygroscopicity

1 g of microcapsules were added into a flat‐bottomed weighing bottle and taken in a suitable drier, at 25 ± 1°C, containing the saturated solution of (NH_4_)_2_SO_4_. After 7 days, the microcapsules were weighed again and hygroscopicity was calculated as the water content per 100 g of microcapsules (Botrel et al., 2012).

#### 2.6.4 Bulk density

2 g of microcapsules were gently added into an empty measuring bottle of 10 ml, and the measuring bottle was vibrated for 1min. The ratio of microcapsule weight to its volume occupied in the measuring bottle was the bulk density (Kha, Nguyen, Roach, & Stathopoulos, 2014).

#### 2.6.5 Scanning Electron Microscope (SEM)

The morphological characteristics were observed by SEM (ZEISS evo 18, Carl Zeiss AG). The samples powder was fixed onto the working stage with double‐faced glue, and a fine gold layer was applied under vacuum. The scanning observation was carried out at 5 kV, with a magnification of 900–1,200×.

#### 2.6.6 Particle size

The laser light diffraction (Masterizer 3000, Malvern Instruments Corp.) was used to determine the particle size of microcapsules. The mean diameter was expressed as d_4,3_ that presented the volume average particle size.

(4) $$d_{4,3}=\frac{\sum n_{i}d_{i}^{4}}{\sum n_{i}d_{i}^{3}}$$

Where *n_i_* represents the number of particles with diameter *d_i_*.

The dispersity of microcapsules was characterized by the span value, calculated according to Equation (5).

(5) $$\text{span}=\frac{d_{90}‐d_{10}}{d_{50}}$$

Where *d*_10_, *d*_50_, and *d*_90_ corresponded to 10, 50, and 90 vol% of microcapsule diameters, respectively, on the relative cumulative dimensional distribution curve.

#### 2.6.7 Fatty acid analysis

For the fatty acid analysis of encapsulated and unencapsulated SBPO, the methyl esterification of samples was performed by boron trifluoride–methanol method (Moigradean, Poiana, Alda, & Gogoasa, 2013). The chromatography‐mass spectrometry (GC‐MS) (Thermo Trace 1300‐ISQ QD, Thermo Fisher Scientific Inc., USA) was used to the analysis of the relative percentage of fatty acids. TG‐5MS capillary column (30 m × 0.25 µm × 0.32 mm) was used, and helium (purity 99.99%) was taken as the carrier gas at a flow rate of 1.5 ml/min and a split ratio of 1:30. The warm‐up procedure was as follows: the initial temperature was set at 80°C and increased to 150°C at the rate of 20°C/min for a hold of 1 min. Then, it was increased to 280°C at the rate of 3°C/min for a hold of 10 min. The injector temperature was 280°C. MS spectrum was analyzed in full‐scan mode with the mass range of 33–550 amu and the ionization voltage was 70 eV.

#### 2.6.8 Peroxide value (PV)

The SBPO microcapsules and SBPO were placed in an oven at 60°C to accelerate the oxidation process, and the PV was measured weekly for 4 weeks. 30 g of microcapsules was used to extract the wrapped oil by the following method: Firstly, the microcapsules were placed in a breaker with 300 ml of hexane and the sample was kept a vortex movement for 1 min to remove the surface oil of microcapsules. Then, the solvent was filtered and the microcapsules were air‐dried in the shade and then treated by the same procedure described previously for the extraction of the total oil. The PV of oil was determined referring to the method described by Karaca, Low, and Nickerson (2013), with some modification.

2 g of oil was placed in a 250 ml of iodine flask and 30 ml of chloroform/glacial acetic acid mixture (in 2:3, v/v) was added, and gently shaken to dissolve the sample completely. Then, 1 ml of saturated potassium iodide solution was added, shaken gently for 30 s, and left in the dark. After 3 min, 100 ml of distilled water and 1ml of starch indicator solution (1 g/100 ml water) were added. The sodium thiosulfate solution (0.01 mol/L) was used to titrate the mixture until the blue color disappeared. A blank test was also performed. The following equation was used to calculate PV:

(6) $$\text{PV}=\frac{\left(V‐V_{0}\right)\times c}{2\times m}\times 1000.$$

Where *V* is the volume of sodium thiosulfate titrated in the sample (mL), *V*_0_ is the volume of sodium thiosulfate titrated in the blank (mL), *c* is the concentration of sodium thiosulfate solution (0.01mol/L), and *m* is the sample weight (g).

### 2.7 Statistical analysis

All the experiments repeated three times. The data were analyzed statically using Design‐Expert 8.0.6 procedure (State‐Ease, Inc.). The calculation and simulation of the optimal operation conditions were carried out by an analysis of variance (ANOVA).

## 3 RESULTS AND DISCUSSION

### 3.1 Single‐factor experiments

**FIGURE 1:**
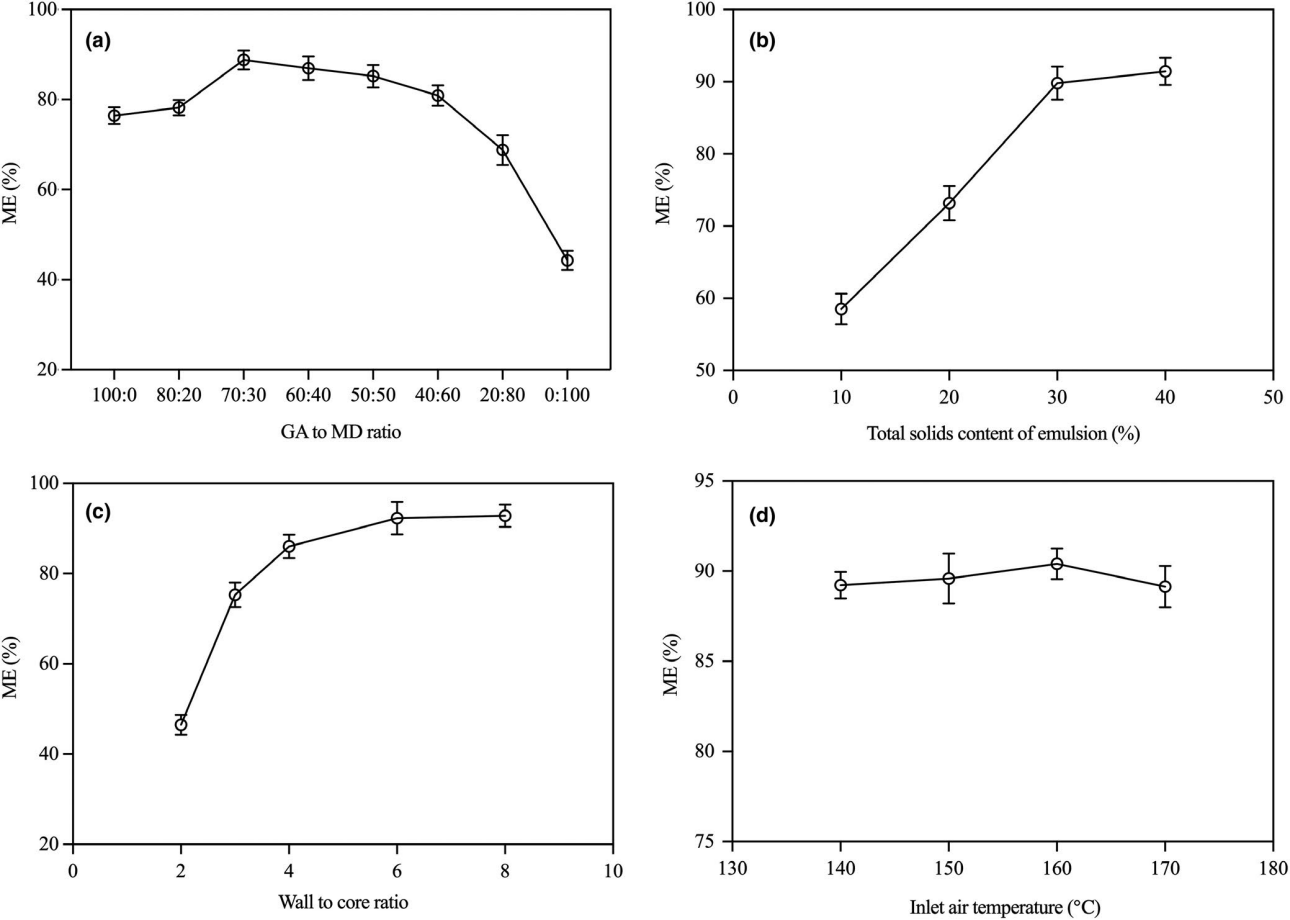
Single‐factor experiments. (a) Effect of GA to MD ratio on ME. Total solids content 30%, wall to core ratio 4, inlet air temperature 160°C, outlet air temperature 80 ± 5°C; (b) effect of total solids content of emulsion on ME. GA to MD ratio 70:30, wall to core ratio 4, inlet air temperature 160°C, outlet air temperature 80 ± 5°C; (c) effect of wall to core ratio on ME. GA to MD ratio 60:40, total solids content 30%, inlet air temperature 160°C, and outlet air temperature 80 ± 5°C. (d) Effect of inlet air temperature on ME. GA to MD ratio 60:40, total solids content 30%, wall to core ratio 4, outlet air temperature 80 ± 5°C

The effect of different ratios of GA to MD on ME was studied as shown in Figure 1a. When the GA to MD ratio was 100:0, ME was 76.45%. With the proportion of MD in wall material increased from 0% to 30%, ME was increased from 76.45% to 88.80%. However, ME continued to decrease once the proportion of MD was more than 30%. The ME was only 44.30% when MD was used as the only wall material. The structure of GA was a highly propped hybrid of glucuronic acid, sugars and a small amount of protein that was covalently linked to the carbohydrate chain through serine and hydroxyproline residues, making it a special emulsifier and film‐forming (Rosenberg et al., 1990). So the ME was satisfactory when GA was used as the only wall material. The similar result was reported by some researches (Bertolini et al., 2001; Fang et al., 2005; McNamee et al., 2001). The fact that ME was the lowest when MD was used as the only wall material could be explained as a result of the weak emulsification and film formation of MD. The results showed that the blend of GA and MD could improve the ME of sea buckthorn pulp oil, but the proportion of MD should be control in an appropriate extent.

It was shown in Figure 1b that the ME was also affected by total solids content of emulsion. Blow 30% solids, particularly, as the solid content increases, the value of ME increased dramatically. When more than 30%, the increased degree was not markedly. The speed of forming the semi‐permeable membrane was accelerated by increasing the solids of emulsion, which resulted in the reduction of the feed loss (Soottitantawat et al., 2005). The similar phenomenon was also recognized by some researchers (Eccleston et al., 2002; Kallio, Yang, Peippo, Tahvonen, & Pan, 2002; Roccia, Martínez, Llabot, & Ribotta, 2014; Yang et al., 1999). Rosenberg et al. (1990) recommended that the highest total solids content should be used, while some results showed that a suitable total solids content was practical (Gallardo et al., 2013; Gharsallaoui et al., 2007; Pitalua et al., 2010; Roccia et al., 2014). Beyond some total solids content, excessive wall materials would not be dissolved due to the limited solubility of wall materials, which made the undissolved wall materials being useless. At the same time, a high emulsion viscosity would be induced with high total solids content, making some difficulties in feeding and atomizing. So there was an optimum total solids content for spray drying.

As shown in Figure 1c, increasing the wall material content in emulsion could significantly increase the ME. The ME increased from 46.47% to 86.05% with the ratio of wall to core increased from 2 to 4. At the low ratio of wall to oil, the wall material could not effectively wrap more oil, causing some oil to remain on the surface of the microcapsules. At the same time, the low content of wall material may also result in an increase in shell forming time during drying process. With the increasing of wall to core ratio, more oil was sufficiently encapsulated by more wall material, resulting to the increasing of ME. However, the ME was not increased significantly on further increasing wall to core ratio to 6 and 8. Kha et al. (2014) detailedly studied the effect of wall material concentration and oil load on the qualities of gac oil microcapsules by spray drying. The results showed that the ME and encapsulation yield were increased when the gac oil load decreased. Meanwhile, the PV, moisture content, and water activity of gac oil microcapsules were all decreased with the increase of wall material concentration. In the microencapsulation of flaxseed oil by spray drying, Tonon et al. (2012) also found that the increase in oil concentration directly affected powder properties, resulting in lower ME and higher lipid oxidation. The ME of fish oil microcapsules by spray drying was decreased from 92.10% to 57.39% when the ratio of wall to oil decreased from 2 to 0.67(Tan, Chan, & Heng, 2005). Therefore, the ratio of wall to core also had important effect on the qualities of product during spray drying.

As shown in Figure 1d, the ME slightly increased from 89.23% to 90.41% with inlet air temperature increasing from 140 to 160°C. When inlet air temperature was further increased to 170°C, the ME slightly decreased to 89.15%, which might have resulted from bursting of the droplet during spray drying. Generally, the ME increased with the increasing of inlet air temperature, the similar phenomenon was also reported by other researchers (Aghbashlo, Mobli, Madadlou, & Rafiee, 2013; Huang et al., 2014; Kalkan, Vanga, Murugesan, Orsat, & Raghavan, 2017; Roccia et al., 2014; Shamaei, Seiiedlou, Aghbashlo, Tsotsas, & Kharaghani, 2017). The high temperature could accelerate the evaporation of water and lead to the rapid formation of a hard crust on the droplet surface. Once the hard crust was formed around the particles, it produced a protective effect similar to that of a semi‐permeable membrane and prevented the further leaching of oil droplet to the particle surface, leading to a high ME. When the temperature was too low, the poor microcapsules, with the characteristics of easy clumping, high in water content, and density membrane, would be more prone to appear, caused by the low evaporation rate. However, if the temperature was too high, some bad states would be occurred, such as thermal damage, “ballooning,” excessive bubble growth, and surface defect (Jafari et al., 2008; Shahidi & Han, 1993).

Meanwhile, inlet air temperature could also affect produced yield and other properties of microcapsules, such as moisture content, water activity, hygroscopicity, color differences, particle size, bulk density, and glass transition temperature. Generally, in the appropriate temperature range, the moisture content and water activity of spray‐dried products decreased with the increasing of inlet air temperature due to high heat transfer and the higher color differences were obtained under higher temperature because of the non‐enzymatic browning reactions. The bigger particles were easily produced under high temperature, which was often contributed to the rapid formation of a hard crust on the surface of the droplets hindered the shrinkage of microparticles. However, there were controversial reports on the effect of inlet air temperature on the hygroscopicity, bulk density, and glass transition temperature of products, which might be contributed to the differences in the properties of raw and wall materials, processing conditions and so on. The detail reports and analysis had been reviewed by Tontul and Topuz (2017), Shishir and Chen (2017). Therefore, the inlet air temperature was a very critical factor in the spray‐drying process. It was important to choose the appropriate inlet air temperature for good quality product.

### 3.2 Optimization of spray‐drying process by RSM

#### 3.2.1 Fitting the mathematical model and statistical analysis

The experiment data and predicted data were listed in Table 1. The fitted model equation was given as followed:

(7) $$\begin{array}{cc} Y= & ‐2087.4358\;+\;53.9677X_{1}\;+\;8.6291X_{2}\;+\;69.2826X_{3}\;+\;22.2876X_{4}‐\\ & \;0.3171X_{1}X_{2}‐1.3993X_{1}X_{3}‐0.2753X_{2}X_{3}‐3.5051X_{1}^{2}‐0.0372X_{2}^{2}‐\\ & \;4.0115X_{3}^{2}‐0.0638X_{4}^{2}. \end{array}$$

Table 2 listed the results of ANOVA. The model with the larger absolute value of *F* and the smaller value of *p* was the more significant. Thus, the value of *F* (*F* = 94.86) and *p* (*p* < .0001) suggested that the model was significant. The value of “lack of fit” *F* (*F* = 5.09) and the associated *p* (*p* = .0655) was not significant, which meant that the model was accurate and applicable. The correlation coefficient (*R* ^2^ = .9896) and adjusted correlation coefficient ($R_{\text{adj}}^{2}$ = 0.9791) also showed the goodness of fitting model. The coefficient of variation (*C.V*.% = 3.28) reflected that the experimental values had a good reliability.

**TABLE 2:** ANOVA of the quadratic model for ME

Source	Sum of squares	DF^a^	Mean square	*F*‐value^b^	*p*‐value	Significance^c^
Model	8,698.11	14	621.29	94.89	<.0001	Significant
X_1_	97.47	1	97.47	14.89	.0017	Significant
X_2_	426.26	1	426.26	65.10	<.0001	Significant
X_3_	5,510.51	1	5,510.51	841.59	<.0001	Significant
X_4_	230.65	1	230.65	35.23	<.0001	Significant
X_1_X_2_	116.21	1	116.21	17.75	.0009	Significant
X_1_X_3_	90.54	1	90.54	13.83	.0023	Significant
X_1_X_4_	20.03	1	20.03	3.06	.1022	Not significant
X_2_X_3_	121.22	1	121.22	18.51	.0007	Significant
X_2_X_4_	24.60	1	24.60	3.76	.0730	Not significant
X_3_X_4_	14.06	1	14.06	2.15	.1649	Not significant
X_1_^2^	665.60	1	665.60	101.65	<.0001	Significant
X_2_^2^	90.00	1	90.00	13.74	.0023	Significant
X_3_^2^	1,670.12	1	1,670.12	255.07	<.0001	Significant
X_4_^2^	264.33	1	264.33	40.37	<.0001	Significant
Residual	91.67	14	6.55			
Lake of fit	84.99	10	8.50	5.09	.0655	Not significant
Pure error	6.68	4	1.67			
Cor total	8,789.78	28				
C.V.%	3.28					
R^2^	0.9896					
adj‐R^2^	0.9791					

^a^ Degrees of freedom.

^b^ Test for comparing term variance with residual value.

^c^ *p* < .05 significant.

It also was shown in Table 2 that the factors that had significant effects on the ME were linear coefficients of *X*_1_, *X*_2_, *X*_3_, and *X*_4_, interaction coefficients of *X*_1_ *X*_2_, *X*_1_ *X*_3_, and *X*_2_ *X*_3_, and quadratic coefficients of $X_{1}^{2}$, $X_{2}^{2}$, $X_{3}^{2}$, and $X_{4}^{2}$. The other coefficients (*X*_2_ *X*_4_ and *X*_3_ *X*_4_) were not significant (*p* > .05).

#### 3.2.2 Analysis of response surface model

**FIGURE 2:**
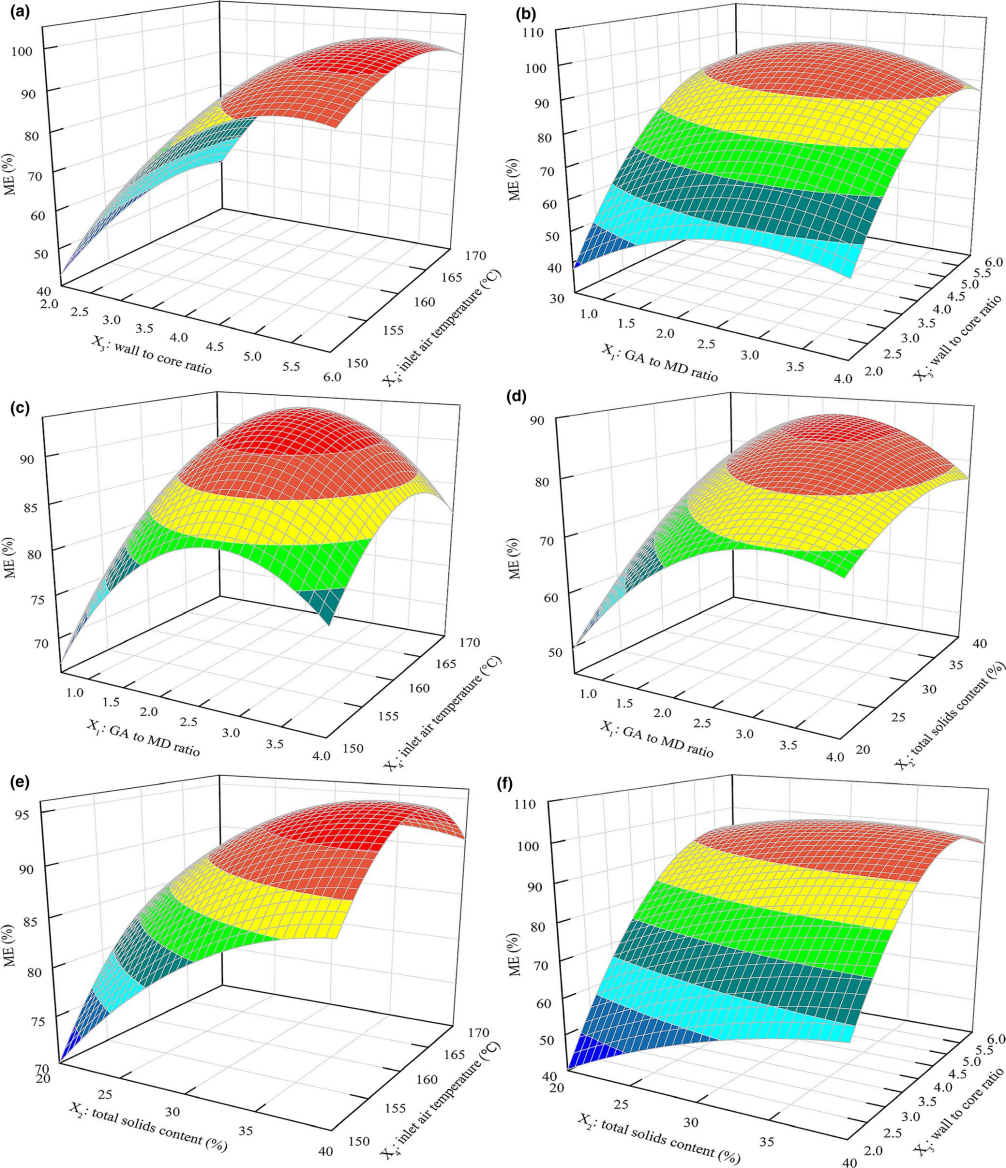
The 3D response surface plots. (a) Wall to core ratio and inlet air temperature; (b) GA to MD ratio and wall to core ratio; (c) GA to MD ratio and inlet air temperature; (d) GA to MD ratio and total solids content; (e) total solids content and inlet air temperature; (f) total solids content and wall to core ratio

The visual interactions among tested variables were presented in Figure 2. As exhibited in Figure 2a, wall to core ratio and inlet air temperature had a significant effect on the ME, which demonstrated that the suitable wall to core ratio and inlet air temperature were in favor of the ME. As presented in Figure 2b, the ME was given as a function of GA to MD ratio and wall to core ratio when total solids content was fixed at 30% and inlet air temperature at 160°C. At a fixed GA to MD ratio, the ME increased significantly with increasing wall to core ratio until the value of 4 was reached after which the trend of increasing became less significant. Figure 2c showed the interactions between GA to MD ratio and inlet air temperature when total solids content was fixed at 30% and wall to core ratio at 4. At a fixed GA to MD ratio, the ME initially increased and then declined with increasing inlet air temperature. A higher inlet air temperature caused an excessive evaporation and resulted in the crack of microcapsules during spray drying. Figure 2d also showed that the ME initially increased and then declined with increasing GA to MD ratio. As observed in Figure 2e,f, at a given inlet air temperature or wall to core ratio, the ME initially increased and then became stabilized with increasing total solids content.

Based on the Equation (7), the optimal experimental conditions were as follows: GA to MD ratio 2.38, total solids content 39.08%, wall to core ratio 5.33, and inlet air temperature 153.78°C. Taking into account the actual situation, the experimental conditions were modified slightly as follows: GA to MD ratio 2.38, total solids content 39%, wall to core ratio 5.33, and inlet air temperature 154°C.

#### 3.2.3 Verification of the predictive value

In order to test the accuracy of the predictive model, the validation experiment was conducted under the following conditions: GA to MD ratio 2.38, total solids content 39%, wall to core ratio 5.33, and inlet air temperature 154°C. The experimental value of ME was 94.96 ± 1.42% and very close to the predicted value (95.96%). The results suggested that the optimized conditions obtained from the mathematical model were reliable and practical.

### 3.3 Properties of microcapsules

For oil powder, the bad phenomenon of oil oxidation and microbiological growth can occur more easily with high water activity and moisture content. As seen in Table 3, the value of water activity and moisture content for microcapsules was 0.44 and 3.18%, respectively, which can be considered quite microbiological stable during storage. The microcapsules had considerable solubility, with a WSI value of 85.56%, due to the excellent water solubility of MD and GA, which were often used as wall materials in the spray‐drying process (Quek, Chok, & Swedlund, 2007; Tan et al., 2015).

**TABLE 3:** The physical properties of microcapsules

Physical properties	
Water activity	0.44 ± 0.02
Moisture content (%)	3.18 ± 0.18
WSI (%)	86.56 ± 0.91
Hygroscopicity (%)	26.67 ± 0.35
Bulk density (g/cm^3^)	0.48 ± 0.02

The hygroscopicity refers to water absorption performance of the substance under certain temperature and humidity conditions. Generally, the lower the hygroscopicity of a substance, the higher its stability. The hygroscopicity of sea buckthorn pulp oil microcapsules was 26.67%, which was similar to the hygroscopicity of oregano essential oil microcapsules (Botrel et al., 2012). Kurozawa, Morassi, Vanzo, Park, and Hubinger (2009) found that the hygroscopicity of microcapsules could be decreased by increasing the concentration of MD.

The bulk density of microcapsules was 0.48 g/cm^3^, which was similar to the encapsulated flaxseed oil (Carneiro, Tonon, Grosso, & Hubinger, 2013), oregano essential oil (Botrel et al., 2012), and gac oil (Tuyen, Nguyen, & Roach, 2010). Generally, the power with high bulk density is required because of the need for smaller volumes of packaging. Moreover, the high bulk density suggested a low amount of air inhaled in the space between particles, which helped prevent oxidation and improve stability.

**FIGURE 3:**
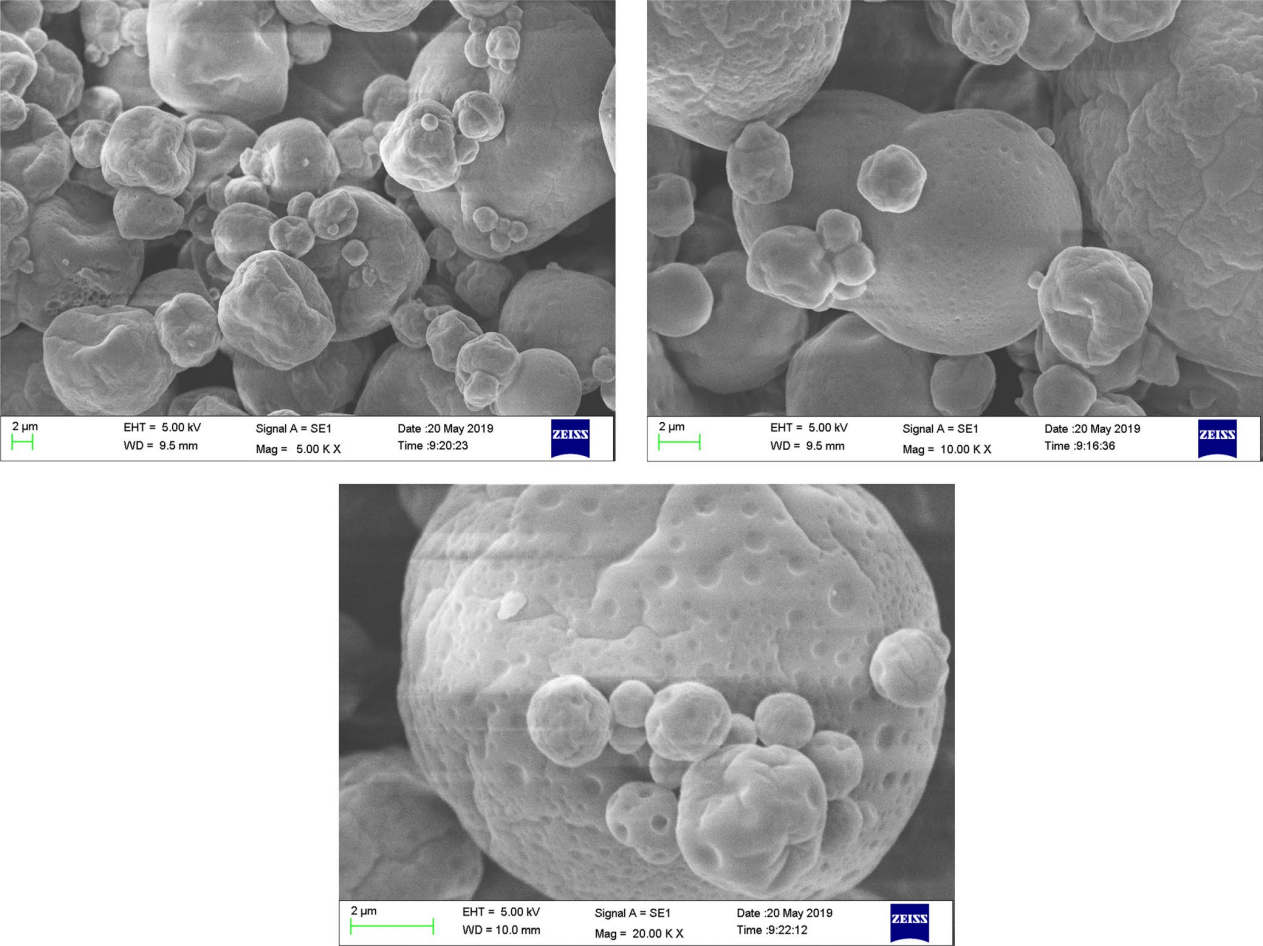
Microphotograph of SBPO microcapsules

**FIGURE 4:**
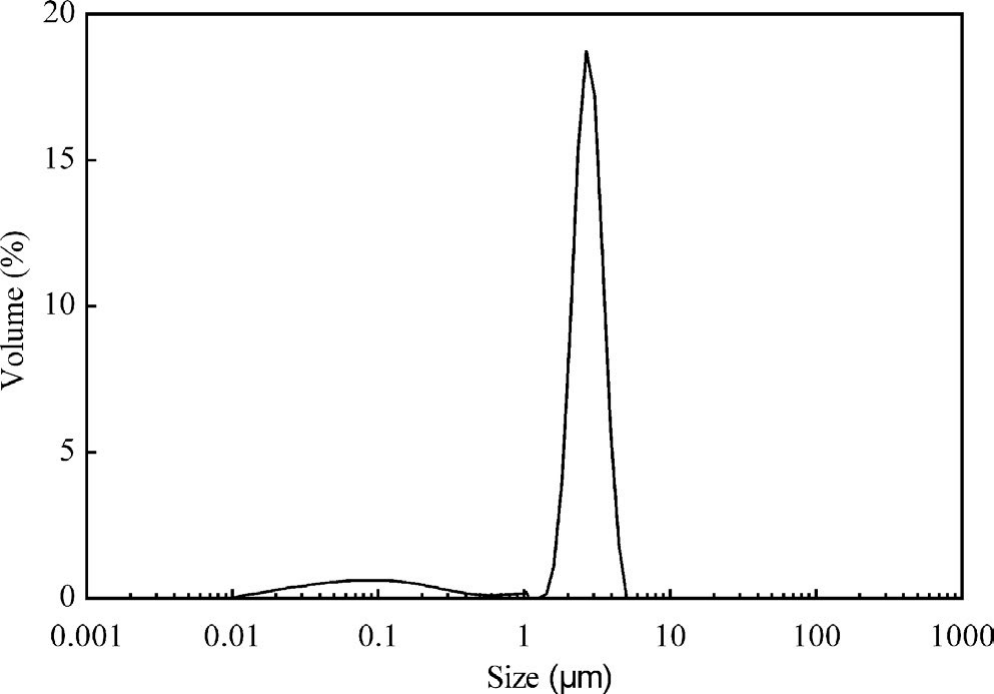
Particle size distribution of SBPO microcapsules

The morphology of microcapsules was shown in Figure 3. As seen from Figure 3, there were microcapsules with different morphological features, including round particles and particles with surface dents. It was also shown that the microcapsules with surface dents occupied a large proportion. Many studies had confirmed that the drying temperature taken an important effect on the morphology of microcapsules (Janiszewska & Witrowa‐Rajchert, 2009; Mestry, Mujumdar, & Thorat, 2011; Nijdam & Langrish, 2006; Tan et al., 2015; Tonon, Brabet, & Hubinger, 2008). At high drying temperatures, the moisture of the particles evaporated quickly, resulting to most of particles having a smooth and hard outer shell. When the drying temperature was low, more residual moisture made the surface of the particles soft, the particles tended to have a shrinking surface. Cracks and pores were not observed in most microcapsules, which was beneficial for the protection of compounds in microcapsules. It was shown in Figure 4 that the particle size of microcapsules ranged from 0.01 to 5 μm. The *d*_10_, *d*_50_, and *d*_90_ were 0.19, 2.77, and 3.82 μm, respectively. The mean diameter *d*_4,3_ was 2.55 μm, and the span value was 1.38. The results showed that the particle size distribution of microcapsules was narrow.

The main fatty acids of unencapsulated and encapsulated SBPO in the term of peak area (%) were present in Table 4. Oleic acid was the highest concentration in the unencapsulated and encapsulated SBPO. The concentration of oleic acid reduced from 21.23% to 18.40% after microencapsulation, which could be due to oxidation during the spray‐drying process. The concentration of palmitoleic acid, palmitic acid, and linoleic acid also showed a decreasing trend. However, on the whole, there were no significant changes in the content of the main fatty acids in SBPO before and after spray drying. Wan, Bankston, Bechtel, and Sathivel (2011) reported that the content of total saturated fatty acids and monounsaturated fatty acids of menhaden fish oil had a slightly increasing after encapsulation by spray drying with soluble rice bran fiber as wall material. Compared to unencapsulated menhaden fish oil, the content of eicosapentaenoic acid and docosahexaenoic acid in the encapsulated menhaden oil was reduced from 14.49% to 11.52% and from 6.15% to 4.51%, respectively. The similar results were also reported by Lavanya, Kathiravan, Moses, and Anandharamakrishnan (2019) in the microencapsulation of fish oil by spray drying with whey protein as wall material. Adamiec and Kalemba (2006) compared the changes in compositions of elemi oil and peppermint oil after microencapsulation by spray drying with maltodextrin as wall material. After microencapsulation, the proportion of the main components in elemi oil including α‐thujene, α‐pinene, β‐pinene, p‐cymene, and γ‐terpinene reduced by 10%–30% and the proportion of the main components including sabinene and terpinene‐4‐ol increased 7% and 62%, respectively. However, the proportion of all major components in peppermint oil including menthone, isomenthone, neomenthol, menthol, menthyl acetate, and monoterpene hydrocarbons differed only slightly from the initial oil after microencapsulation. In the process of oil microencapsulation by spray drying, the degree of change of components was influenced by several factors, such as inlet air temperature, properties of wall materials, and self‐thermal stability of components. Therefore, it was important to select appropriate wall materials and operating parameters to reduce the loss of ingredients.

**TABLE 4:** The main fatty acids of unencapsulated and encapsulated SBPO

Fatty acid composition	Fatty acid (%)	
	Unencapsulated SBPO	Encapsulated SBPO
Palmitoleic acid	10.62 ± 0.08	10.44 ± 0.09
Palmitic acid	12.77 ± 0.11	12.17 ± 0.13
Linoleic acid	5.67 ± 0.05	3.84 ± 0.04
Oleic acid	21.23 ± 0.24	18.40 ± 0.15
Stearic acid	2.76 ± 0.02	2.91 ± 0.03
cis‐11‐Octadecenoic acid	2.51 ± 0.01	3.25 ± 0.02

**FIGURE 5:**
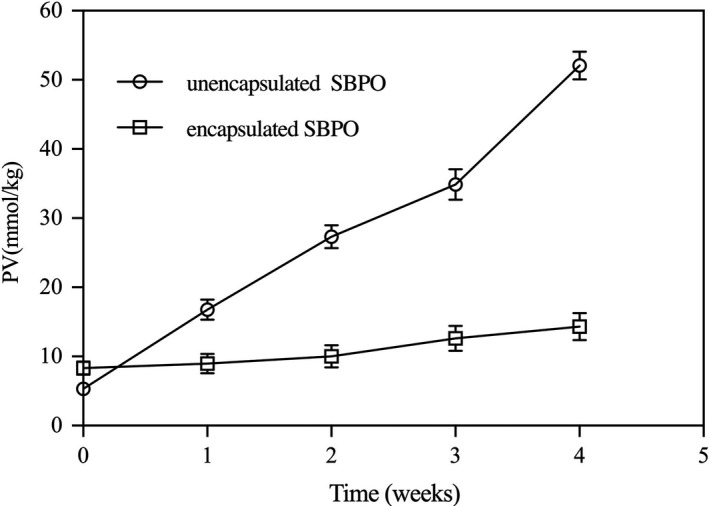
Changes in PV of unencapsulated and encapsulated SBPO under 60°C

Peroxide value was an important indicator of the oxidation degree of oils. The PV of products made from oils was used to identify the quality and degree of spoilage. Changes in PV of unencapsulated and encapsulated SBPO under 60°C were shown in Figure 5. The initial PV of encapsulated SBPO was higher than that of unencapsulated SBPO, which indicated that spray drying increased the PV of SBPO. A similar phenomenon was also observed in the microencapsulation process of other oils by spray drying, such as crude palm oil (Ferreira et al., 2016), fish oil (Aghbashlo et al., 2013; Lavanya et al., 2019), chia oil (Lavanya et al., 2019), almond oil (Hoyos‐Leyva, Bello‐Perez, Agama‐Acevedo, Alvarez‐Ramirez, & Jaramillo‐Echeverry, 2019), and flaxseed oil (Can Karaca, Low, & Nickerson, 2013). The increase in PV was often attributed to high temperatures during spray drying. During the high‐temperature acceleration experiment for 4 weeks, the PV of unencapsulated SBPO increased from 5.31 to 52.08 mmol/kg, while the PV of encapsulated SBPO increased from 8.31 to 14.34 mmol/kg. The results presented that the microencapsulation could effectively protect SBPO from oxidation.

## 4 CONCLUSION

In this work, SBPO microcapsules were produced by spray drying. Based on the single‐factor experiment, RSM was used to optimize the operating conditions including GA to MD ratio, total solids content of emulsion, wall to core ratio, and inlet air temperature. The optimal conditions were as follows: GA to MD ratio 2.38, total solids content 39%, wall to core ratio 5.33, and inlet air temperature 154°C. Under the optimal conditions, the ME of microcapsules was 94.96 ± 1.42%, which was satisfactory for the quality requirements of oil microcapsules. The physicochemical properties presented that SBPO microcapsules had low water activity, low moisture content, high water solubility, high bulk density, and good thermal stability, which were useful for subsequent processing.

## CONFLICT OF INTEREST

There are no conflicts of interest.

## ETHICAL APPROVAL

This study does not involve any human or animal testing.
